# Intermittent preventive treatment for the prevention of malaria during pregnancy in high transmission areas

**DOI:** 10.1186/1475-2875-6-160

**Published:** 2007-12-04

**Authors:** Valérie Briand, Gilles Cottrell, Achille Massougbodji, Michel Cot

**Affiliations:** 1 Mother and Child Health in the Tropics (UR010), Development Research Institute (IRD), Paris, France; 2 Parasitology and Mycology education and research unit (UER), Science and Health Faculty (FSS), Cotonou, Benin

## Abstract

Malaria in pregnancy is one of the major causes of maternal morbidity and adverse birth outcomes. In high transmission areas, its prevention has recently changed, moving from a weekly or bimonthly chemoprophylaxis to intermittent preventive treatment (IPTp). IPTp consists in the administration of a single curative dose of an efficacious anti-malarial drug at least twice during pregnancy – regardless of whether the woman is infected or not. The drug is administered under supervision during antenatal care visits. Sulphadoxine-pyrimethamine (SP) is the drug currently recommended by the WHO. While SP-IPTp seems an adequate strategy, there are many issues still to be explored to optimize it. This paper reviewed data on IPTp efficacy and discussed how to improve it. In particular, the determination of both the optimal number of doses and time of administration of the drug is essential, and this has not yet been done. As both foetal growth and deleterious effects of malaria are maximum in late pregnancy women should particularly be protected during this period. Monitoring of IPTp efficacy should be applied to all women, and not only to primi- and secondigravidae, as it has not been definitively established that multigravidae are not at risk for malaria morbidity and mortality. In HIV-positive women, there is an urgent need for specific information on drug administration patterns (need for higher doses, possible interference with sulpha-based prophylaxis of opportunistic infections). Because of the growing level of resistance of parasites to SP, alternative drugs for IPTp are urgently needed. Mefloquine is presently one of the most attractive options because of its long half life, high efficacy in sub-Saharan Africa and safety during pregnancy. Also, efforts should be made to increase IPTp coverage by improving the practices of health care workers, the motivation of women and their perception of malaria complications in pregnancy. Because IPTp is not applicable in early pregnancy, which is a period when malaria may also be deleterious for women and their offspring, there is a necessity to integrate this strategy with other preventive measures which can be applied earlier in pregnancy such as insecticide-treated nets.

## Background

For a long time, malaria in pregnancy has been associated with a range of deleterious effects in women and their offspring. It has been recognized as a public health priority since the beginning of the 1980s. Currently, it is estimated that each year more than 25 million women become pregnant in malaria endemic areas – mostly in sub-Saharan Africa – and 75,000 to 200,000 infant deaths are attributable to malaria infection in pregnancy [1,2].

The severity of clinical manifestations is determined by the level of immunity before pregnancy, which depends on the intensity and stability of malaria transmission. In low and/or unstable transmission areas, the degree of acquired immunity of women prior to pregnancy is low, and both the mother and her foetus are at risk for the most severe consequences of the infection. Malaria infections are symptomatic, and the current strategy is to treat every malarial access effectively. In contrast, in areas of high malaria transmission (as in sub-Saharan Africa), women have acquired a protective immunity prior to pregnancy. Malaria infections are generally asymptomatic and the strategy is based on the prevention of infections. Since the early 2000's, prevention has been based on intermittent preventive treatment (IPTp) and insecticide-treated bed nets (ITNs) [1].

The introduction of IPTp was a turning point in the prevention of malaria during pregnancy in high transmission areas. This paper aimed to critically review available data on its efficacy and effectiveness.

### Consequences of malaria in pregnancy

In high transmission areas, malaria is associated with maternal anaemia – potentially responsible for maternal death when severe – and low birth weight (LBW) due to both prematurity and intrauterine growth retardation [3]. LBW is a high risk factor for perinatal death and it is also correlated with morbidity and mortality during infancy [4,5]. A recent study estimated that malaria may contribute to 3–5% of maternal anaemia, 8–14% of LBW, and 3–8% of infant mortality [2]. Malaria consequences are particularly deleterious in women co-infected with HIV, as they have more frequently clinical and placental malaria, more detectable malaria parasitaemia and higher malaria parasite densities. HIV-positive women of all gravidities are at increased risk, although primi- and secundigravidae are the most affected overall [6].

Malaria during pregnancy may also influence the development of antimalarial immunity during the first years of life. Infants born to placenta-infected mothers were shown to be more likely to develop a malaria infection between four and six months of life, compared to those born to non-infected mothers [7]. Recently, similar results were obtained in a different malaria-endemic area [8]. It was suggested that offspring of placental-infected multi gravid women had the highest risk of parasitaemia during the first years of life compared to children of primigravid and/or placental non-infected women.

### Strategies to prevent malaria during pregnancy

In Africa, the first malaria preventive strategies were implemented in the 1950s. They consisted in weekly or bi-monthly chemoprophylaxis with chloroquine (CQ) in West African countries and dapsone-pyrimethamine or sulphadoxine-pyrimethamine (SP) in East African countries. A large number of trials demonstrated the efficacy of such a chemoprophylaxis in preventing LBW, maternal anaemia and placental malaria infection [9,10]. Unfortunately, because of the growing resistance of parasites to these drugs and the poor compliance of the women with the treatment the strategies finally showed a low efficacy. In 1998 it was proposed, then finally implemented in 2004, that chemoprophylaxis should no longer be recommended, but replaced by intermittent preventive treatment for all pregnant women living in areas of stable malaria transmission [1].

IPTp consists in the administration of a single curative dose of an efficacious anti-malarial drug at least twice during pregnancy – regardless whether or not the woman is infected. The drug is administered under supervision during antenatal care (ANC) visits. Sulphadoxine-pyrimethamine is the drug currently recommended by the WHO because of its safety and efficacy in pregnancy [1]. Several studies have shown the high efficacy of IPTp with SP, compared to placebo or CQ prophylaxis, on placental infection, LBW and/or severe maternal anaemia [11-19].

## IPTp with sulphadoxine-pyrimethamine: efficacy and effectiveness

While IPTp has been shown to be a promising strategy in terms of efficacy and acceptability, some issues should be adressed.

### How many IPTp doses should women receive?

WHO recommends the administration of at least two doses of SP during pregnancy [1]. This recommendation was based on the average number of ANC visits women had in African countries, and on the results of the first IPTp studies [11,12,16]. While two doses were found more efficacious than a single dose [16-20], few studies have investigated the efficacy of a higher number of intakes. These studies showed that three or more SP doses were more efficacious than two in HIV-positive women, but that no benefit was found in HIV-negative women [16,18,21]. However, in all these studies but one [16] the results might have been biased as the number of doses the women received was not randomized but depended on how often the women attended for ANC visits.

Hence, the efficacy of a higher number of IPTp doses is still to be evaluated both in HIV-negative and HIV-positive women. These evaluations should be done in the context of ITN use. ITN is the only strategy to be used during the first trimester of pregnancy, when malaria may also be deleterious for women and their offspring [22]. IPTp is not applicable at that time, because most antimalarial drugs are contraindicated (due to possible toxicity on the foetus) and most women do not attend ANC visits. In addition to their additive effect [23], it was suggested that the ITN and IPTp strategies could even be synergistic [13].

Also, the optimal number of doses should be evaluated in terms of applicability. IPTp coverage with a full two-dose treatment, which is the strategy recommended by almost all countries with an IPTp policy, is still low. The administration of a higher number of doses may be difficult to implement. Further studies are required to assess the safety of administering more doses of SP in pregnant women [16,21].

### When should women receive IPTp?

WHO recommends that the first dose should be administered at the first ANC visit after quickening – which ensures that the woman is in the second trimester of pregnancy [1]. Following IPTp doses should be given at least one month apart. There is currently no precision regarding the best timing for their administration as it entirely depends on the timing and frequency of ANC visits of the woman. When applicable, women should particularly be protected in late pregnancy, when both foetal growth [24] and deleterious effects of malaria are most important [23,25]. In the absence of ANC visits in the two first trimesters, it could still be worthwhile to administer IPTp even only in the last month of pregnancy. The baby is still growing and has to be protected, and there is no major contraindication in using SP close to delivery. A single study found an increased risk of kernicterus in neonates treated with sulphonamides [26], but this has not been further confirmed [27]. Two recent studies highlighted the relevance of a protection in late pregnancy [19,21]. Van Eijk *et al *found that IPTp had a higher efficacy when the last dose was administered close to delivery [19]. Similarly, Filler *et al *showed a better protection in women randomized in a SP-monthly group, who received their last dose of SP close to delivery, than in women taking the usual two-dose SP-IPTp [21].

The timing of drug administration should be taken into account when evaluating IPTp efficacy. If key moments are identified, women could be strongly encouraged to attend for ANC visits precisely at these times. More fundamental studies are also needed to determine the optimal dosing interval by clarifying how IPTp works (i.e., prophylactic or treatment effect) and by providing pharmacokinetic data in pregnant women for SP [28].

### Which women should receive IPTp?

WHO recommends that pregnant women of all gravidities should receive IPTp [1]. However, most IPTp studies have been limited to primi- and secundigravidae because their offspring have the highest malaria morbidity [29]. A recent study, specifically conducted in multigravidae, did not show any beneficial effect of IPTp on anaemia or LBW in this population of women [30].

The reliability of targeting the intervention to primi- and secundigravidae only is questionable. For equity reasons, it is hardly conceivable to exclude some women from receiving a preventive measure (or public health intervention). When implementing the intervention, it would be difficult to identify precisely the women who have the highest risk (i.e primi- and secundigravidae, plus HIV-infected women of all gravidities), to single them out and protect them specifically. Indeed, obstetrical histories are difficult to collect, especially with regard to events such as abortions or stillbirths, and the HIV status of a woman is generally not known. Furthermore, while malaria has a lower impact in multigravidae, it has not yet been fully assessed how deleterious malaria is in women who have been protected during their first pregnancies [31]. A recent study strengthened the need for multigravidae to be effectively protected as it suggested that their offspring, when the placenta was infected, had a higher risk of parasitaemia during infancy compared to primigravidae [8].

### IPTp effectiveness

While, by 2010, 80% of all pregnant women living in high transmission areas are expected to receive IPTp [32], the coverage of the intervention is still low. In Kenya, one of the first countries to implement IPTp, the national coverage with two doses of SP was only 4% five years after IPTp implementation [33]. Only one country (Malawi) is close to achieving the 2000 Abuja target of 60% coverage of pregnant women by 2005.

WHO advises that IPTp should be administered through ANC services. Although ANC attendance is high in most countries with IPTp policy (median, 2.0–4.8 ANC visits per woman), it has not been sufficient to ensure a high IPTp coverage. Several factors, such as women's perceptions and the practices of health-care workers, have been identified to explain the poor compliance with IPTp [33,34]. As an example, in an ongoing clinical trial conducted in Benin, which aims to compare the efficacy of SP versus mefloquine (MQ) for IPTp [35], it was found that 30% of the women did not attend the maternity clinic to receive the second dose, and 40% continued to receive CQ during the first trimester although the IPTp strategy has been extensively explained to the women and midwives participating to the trial (Briand, personal communication).

IPTp has been shown to be an efficacious strategy. However, a theoretically efficacious measure is not worthwhile if it is not well applied in the field. Anthropological studies and knowledge, attitudes and practices surveys should help to improve the implementation of IPTp by improving the understanding of women's and the motivation of health-care workers. Furthermore, pragmatic studies are required to assess the efficacy of IPTp in "real life" conditions.

## Should malaria prevention strategies change?

Because of the growing level of resistance of parasites to SP, there are concerns about how long IPTp with SP, as currently recommended by the WHO, will remain useful. Resistance to SP has been first described several years ago in East-Africa, and it is rapidly spreading in West Africa [36]. In Benin, where IPTp has recently been implemented, the resistance rate to SP was found to be around 50% in children under five years of age living in a semi-rural area [37].

While it is likely that SP will have soon to be replaced by a more effective antimalarial drug, it is not clear when this change will become necessary. Recently, it was shown that SP could keep its efficacy in public health applications (such as intervention on birthweight or anaemia) in spite of an impaired anti-parasite activity [38]. In the future, the decision of switching to a more efficacious antimalarial drug than SP should be based on the association of several indicators, clinical as well as more directly related to the drug. For this purpose, an active monitoring of pregnant women in selected sentinel sites will probably be necessary.

### Which indicators should be used?

Low birth weight, defined as below 2,500 g, is the main clinical indicator of the consequences of gestational malaria. It is a strong predictor of infant health [4,5], easily measurable and reproducible, so it can be compared between studies and countries. The only problem with LBW is that, being multifactorial, it may require very large samples to evidence differences between groups.

Maternal anaemia is usually determined at the end of pregnancy or at delivery. WHO definitions for mild and severe anaemia are a haemoglobin level below 11 g/dL and 7 g/dL, respectively [39]. Although severe anaemia has been shown to be associated with a higher child and maternal morbidity and mortality, mild anaemia does not seem to confer unquestionably higher risks for the mother or the baby [40]. Hence, severe anaemia should be the best indicator of the two. As the prevalence of severe anaemia is very low, a higher threshold could be used, such as 8 g/dL, as advocated by several authors [12,15].

Placental malarial infection is the main parasitological indicator. Compared to LBW or maternal anaemia, it is not multifactorial and depends only on the presence of parasites. Some authors have suggested to check for peripheral infections during pregnancy instead of placental infection [41]. Nevertheless, placental infection should be more appropriate to evaluate malaria prevention strategies as it is directly linked to the newborn, whom the prevention is focused on. It has been shown to be highly correlated with late peripheral infections, which are the most deleterious for the child [22]. Besides, women can be reached more easily at delivery than during pregnancy. To detect placental infections, thick blood smear are more appropriate than PCR and biopsy. Indeed, it is more easily feasible, cheaper, and a high correlation has been found between placental infection – determined in this way – and low birth weight [3,15,17,42] and prematurity [20]. PCR is more sensitive to detect placental infections, but by being too sensitive it is less discriminating when comparing two drugs or strategies. Biopsy provides additional information regarding chronic and past malaria infections, but these infections are less correlated with birth outcomes, and it is more difficult to perform.

Such clinical indicators are of particular relevance as they have public health implications. However, they do not reflect the efficacy of SP-IPTp only, but the combined efficacy of all the measures that benefit to pregnant women (i.e. IPTp, ITNs and iron and folate supplementation). In order to evaluate the IPTp strategy more specifically, the monitoring of indicators more directly related to the drug (such as *in vivo *SP efficacy) should be done at the same time. *In vivo *therapeutic efficacy of antimalarials is usually evaluated in young symptomatic children. However, resistance rates – determined in this way – poorly correlate with public health delivery outcomes, in particular because of the incomplete immune response to malaria of children compared to adults. In a cross-sectional survey conducted in Benin, CQ was shown to be still highly efficacious to prevent LBW, while the resistance to the drug was up to 90% in the children under five years of age living in the same area [43]. Also, a recent study discussed the relevance of extrapolating data collected in children to pregnant women, as both groups showed different sensitivity to drugs such as CQ or SP [44]. To collect more meaningful data, *in vivo *SP efficacy should be determined in pregnant women rather than children. In parallel, the correlation of this protection with molecular markers of SP resistance should be investigated.

### Which alternative drugs for IPTp?

In addition, there is a need to evaluate alternative antimalarial drugs for IPTp, in terms of efficacy, tolerability and acceptability, to replace SP when it is considered not efficacious enough to protect women.

Ideal properties for an alternative drug are: (1) having a long-half life, as it has been suggested that IPTp had a prophylactic rather than a treatment effect and that the duration of prophylaxis was the most important determinant of IPTp efficacy; (2) being safe during pregnancy, and well-tolerated to ensure a high compliance with the treatment in women who are often asymptomatic when infected with malaria; (3) being easy to administer (ideally a single dose); and (4) at an affordable cost.

As recently stated by the WHO, mefloquine is presently one of the most attractive options. It has a long half-life and is still highly efficacious in African countries. Most studies have shown that MQ was safe for use in pregnancy [3,45-47]. A single study has suggested that women who received MQ treatment had a significantly higher risk of stillbirth than women not treated or treated with other antimalarials [48]. Further studies have not confirmed this finding so far [49]. MQ has been associated with a range of side-effects, raising the question of the women's compliance with the treatment. In an ongoing clinical trial, in which Beninese women are randomized to receive SP or MQ (15 mg/kg as a single intake) for IPTp [35], only mild adverse events have been observed – such as vomiting, nausea and dizziness, and they resolved fast (Briand, personal communication). Very few women have refused to take the second dose because of a poor tolerance of the first intake. The drug has been well-accepted in spite of its bitter taste, and there has been less than 1% of early vomiting (within one hour) after giving a fat snack before the women took the drug (as recommended by the manufacturer).

Whatever the alternative drug, the cost will be substantially higher than for SP or CQ. Mefloquine remains expensive even if its cost has recently declined. In a near future, lower cost and higher availability of the drug in African countries should make this option even more realistic.

Artemisinin combination therapies (ACT) are also being evaluated for IPTp. They have been shown to be highly efficacious and safe during pregnancy except when used in the first trimester. However, if the effect of IPTp is mainly prophylactic [28], then short-acting drugs would be expected to provide little benefit. Moreover, ACT are still very expensive and less easily deliverable as they require multiple treatment doses that could not be given as a directly-observed therapy in the ANC clinic. Because the treatment needs to be administered several times, compliance might be low. Other potential candidates, such as SP plus amodiaquine, SP plus azithromycine (keeping in mind the risk of pneumococcus-resistance increase when using azithromycine) and chlorproguanil-dapsone are also being assessed for IPTp. Piperaquine – used in combinations with other antimalarials rather than used alone – might be one of the most promising options for IPTp.

## Conclusion

There is evidence that IPTp with SP is presently the most efficacious and adequate strategy for the prevention of malaria during pregnancy in high transmission areas. However, several years after having been implemented various problems have to be solved. In particular, it seems that only some of the women exposed to malaria are benefiting from this policy. Efforts should be made to improve performance of health worker and the motivation of women and their perception of malaria complications in pregnancy. The growing resistance of parasites to SP requires an urgent evaluation of alternative drugs to SP. Some attention should be given to the optimal dosage and frequency of administration, which have never been clearly determined. Finally, as IPTp is not applicable in early pregnancy – as most antimalarial drugs are contraindicated during the first trimester – one should continue combining the strategy with ITNs which can be applied earlier in pregnancy, and thus could add to late pregnancy's measures such as IPTp.

## Competing interests

The author(s) declare that they have no competing interests.

## Authors' contributions

VB drafted the manuscript and participated in the design and coordination of the ongoing clinical trial conducted that is discussed in the manuscript; GC helped to draft the manuscript; AM participated in the design and coordination of the ongoing clinical trial; MC conceived of the clinical trial, and participated in its design and coordination and helped to draft the manuscript. All authors read and approved the final manuscript.
